# Incidence and predictors of self-reported pulmonary exacerbations in primary ciliary dyskinesia: an international prospective cohort study

**DOI:** 10.1183/23120541.00686-2025

**Published:** 2026-01-19

**Authors:** Leonie D. Schreck, Myrofora Goutaki, Eva S.L. Pedersen, Fiona Copeland, Trini López Fernández, Living with PCD patient advisory group, Jane S. Lucas, Claudia E. Kuehni

**Affiliations:** 1 Institute of Social and Preventive Medicine, University of Bern, Bern, Switzerland; 2 Graduate School for Health Sciences, University of Bern, Bern, Switzerland; 3 Division of Paediatric Respiratory Medicine and Allergology, Department of Paediatrics, Inselspital, University Hospital, University of Bern, Bern, Switzerland; 4 Department of Clinical Research, University of Bern, Bern, Switzerland; 5 PCD Support UK, Buckingham, UK; 6 Asociación Española de Discinesia Ciliar Primaria/Síndrome de Kartagener, Santo Ángel, Murcia, Spain; 7 Primary Ciliary Dyskinesia Centre, NIHR Biomedical Research Centre, University Hospital Southampton NHS Foundation Trust, Southampton, UK; 8 University of Southampton Faculty of Medicine, School of Clinical and Experimental Medicine, Southampton, UK

## Abstract

**Introduction:**

Pulmonary exacerbations contribute to disease progression in chronic lung diseases. In a large prospective cohort study, we studied the incidence and predictors of pulmonary exacerbations among persons with primary ciliary dyskinesia (PCD), which can inform follow-up care. We also assessed healthcare use, changes in management and pathogens during exacerbations.

**Methods:**

Participants in the Living with PCD study reported increased respiratory symptoms in the past 7 days, indicating a pulmonary exacerbation, from June 2020 through May 2022 *via* online questionnaires. We derived incidence rates and studied predictors of pulmonary exacerbation incidence by fitting multivariable negative binomial regression models.

**Results:**

We obtained data from 660 persons (408 adults, 57 adolescents, 195 children) who completed 17 853 follow-up questionnaires (median 17, range 1–84). The 1026 reported exacerbations indicate an incidence rate of 3.1 pulmonary exacerbations per person per year, with minor variation across age groups, but changes over time. Incidence was higher among adult females (incidence rate ratio (IRR) 2.0, 95% confidence interval (CI) 1.4–2.7) and those in whom *Pseudomonas aeruginosa* was isolated (children IRR 1.9, 95% CI 1.1–3.6; adults/adolescents IRR 1.4, 95% CI 1.0–1.9). Participants saw a health professional during only 185 of 1404 exacerbation weeks (13%). *P. aeruginosa* was the pathogen most frequently observed during exacerbations in children (18 of 118 samples, 15%) and adults/adolescents (132 of 303 samples, 44%).

**Conclusion:**

Pulmonary exacerbations are frequent in PCD and heighten the disease burden. Patients for whom targeted management is particularly important include adult females and those who carry *P. aeruginosa*.

## Shareable abstract

**Pulmonary exacerbations in primary ciliary dyskinesia are frequent, with adult females and people with *Pseudomonas aeruginosa* at higher risk. An international study highlights the need for targeted management to reduce disease burden and improve outcomes.** https://bit.ly/4kLHzvh

## Introduction

Pulmonary exacerbations alter disease management and may contribute to disease progression in primary ciliary dyskinesia (PCD) [1]. Impaired mucociliary clearance leads to recurrent infections, driving more frequent exacerbations, which are likely to result in progressive lung damage and faster deterioration of lung function as in other suppurative lung diseases [2–4]. Exacerbations are primarily triggered by bacterial and viral infections, but host and environmental factors such as pollution and chronic inflammation likely increase the risk of exacerbations [4, 5]. Not all patients recover to baseline lung function after an exacerbation [6, 7], which emphasises the importance of effective prevention and timely management.

Knowing which persons with PCD are at higher risk of exacerbation would enable earlier and more intensive treatment and improve clinical follow-up. However, prospective standardised data on pulmonary exacerbations are scarce. Although azithromycin was found to reduce respiratory exacerbations by 50% compared to placebo in a randomised controlled trial (RCT) [8], data on other factors influencing exacerbation incidence are limited [9–11]. Additionally, inconsistent definitions in previous studies have made comparability difficult, with a consensus definition only recently published [1].

Using data from repeated self-reported questionnaires in a prospective cohort study, we estimated the incidence of pulmonary exacerbations in PCD and studied predictors of higher incidence. We also assessed healthcare use, changes in management and reported pathogens during exacerbations.

## Methods

### Study design and procedure

We studied pulmonary exacerbations using data from the Living with PCD study, an international prospective cohort study initiated in May 2020 by people with PCD and the PCD research team at the University of Bern, Switzerland. The study protocol has been published under its previous name COVID-PCD [12]. All individuals with either a physician-confirmed or suspected diagnosis of PCD were eligible to participate. A physician-confirmed diagnosis was defined as answering “yes” to having been told by a physician that they have, or are likely to have, PCD. A suspected diagnosis was defined as answering “yes” to having reason to believe they have PCD, despite not having received a formal diagnosis from a physician. The initial primary study aim was to monitor health during the coronavirus disease 2019 (COVID-19) pandemic. Participants were invited to take part *via* patient associations worldwide that advertised the study. Participants registered online through the study website (pcd.ispm.ch) and participated anonymously. We collected data through online questionnaires available in five languages (English, French, German, Italian and Spanish) and used the Research Electronic Data Capture (REDCap) database hosted at the Centre for Medical Registries and Data Linkage at the University of Bern, Switzerland [13]. Participants or their caregivers give informed consent when enrolling in the study and can leave the study at any time by informing the study team. The cantonal ethics committee of Bern, Switzerland, has approved the study (study ID: 2020-00830).

### Data collection

All data were self-reported by participants through questionnaires at baseline and through weekly (June 2020 through December 2021) and monthly (January 2022 through May 2022) follow-up questionnaires. The baseline questionnaire covered sociodemographic information, PCD diagnosis, diagnostic tests and results, symptoms and clinical manifestations, isolated pathogens, and antibiotic usage based on modules from the FOLLOW-PCD questionnaire [14]. Supplementary table S1 lists the questions from the baseline questionnaire related to diagnostic testing. The follow-up questionnaires assessed weekly symptoms, healthcare usage, COVID-19 infections and isolation of pathogens.

### Inclusion criteria

This analysis included all participants who completed the baseline and at least one follow-up questionnaire between June 2020 and May 2022.

### Pulmonary exacerbations

Weekly questionnaires assessed new symptoms or increase in chronic respiratory symptoms in the previous 7 days. We covered all items that constituted an exacerbation according to the BEAT-PCD definition [1]. Supplementary table S2 lists the elements of the definition and corresponding questions. We labelled any week in which the definition of a pulmonary exacerbation was met as an exacerbation week. Since exacerbations can last longer than 1 week, we counted consecutive exacerbation weeks as one exacerbation. We only counted a new exacerbation if the participant reported at least one asymptomatic week since the last exacerbation or if no exacerbation week was reported in the last 20 days, assuming that exacerbations usually do not last longer than 4 weeks. We assessed the duration of an exacerbation as the number of consecutive exacerbation weeks. We checked for outliers and implausible values and excluded participants with an implausible proportion of exacerbation weeks (>70% of all weeks) reflecting possible misunderstanding of questions.

### Exposures of interest and other variables

We considered variables previously associated with pulmonary exacerbations or severe airway disease in PCD or other suppurative lung diseases as potential predictors of exacerbation incidence: sex, age, age at diagnosis, affected genes, prophylactic antibiotics, regular physiotherapy, physical activity, body mass index (BMI) and environmental tobacco smoke (ETS). As proxies for disease severity we considered bronchiectasis, congenital heart disease, *Pseudomonas aeruginosa* isolation and forced expiratory volume in 1 s (FEV_1_) [9, 10, 15–17]. All variables were assessed at baseline. Supplementary table S3 defines the variables.

The follow-up questionnaires assessed social contact behaviour categorised as shielding and reduced contacts, and investigated healthcare use and change of therapies during exacerbations (supplementary table S3). We assessed isolated pathogens and positive COVID-19 tests during and outside of exacerbations.

### Statistical analysis

We calculated incidence rates of pulmonary exacerbations per person per year by dividing the number of pulmonary exacerbations by person-years at risk. We defined person-years at risk as the time in years participants were at risk of an exacerbation, assigning 7 days of follow-up to each completed questionnaire and subtracting the time participants already had an exacerbation. We calculated incidence rates stratified by age groups. To assess changes of incidence rate over time, we calculated monthly incidence rates and plotted them together with social contact behaviour (shielding, reduced contacts). We calculated the proportion of exacerbation weeks in all follow-up weeks by dividing the number of exacerbation weeks by the number of completed follow-up questionnaires.

We tested the robustness of the incidence rate calculations in four sensitivity analyses. In the first, we changed the definition of time at risk by not subtracting the time during which participants already had an exacerbation. In the second, we changed the period between two exacerbations to 14 days, and in the third to 30 days, testing the impact of our assumption that exacerbations do not last longer than 4 weeks. In the fourth sensitivity analysis, we only included participants with a physician-confirmed diagnosis.

We studied predictors of higher incidence of pulmonary exacerbations using negative binomial regression analysis with number of exacerbation weeks as outcome and number of completed questionnaires as offset; we chose this model because of the overdispersion in the outcome data. We chose number of exacerbation weeks as outcome to give more weight to longer (*i.e.* more severe) exacerbations. In this analysis, the response “I don't know” for congenital heart disease and bronchiectasis was categorised as “no”, while participants with sex “other” and those with missing values for age at diagnosis and physiotherapy were excluded from the analysis. *A priori* we included sex, age, age at diagnosis, bronchiectasis, congenital heart disease, prophylactic antibiotics and regular physiotherapy in the model and tested all other predictors in univariable analyses, and subsequently also included *P. aeruginosa* isolation since it was strongly associated with incidence. Due to lack of associations in the univariable model, we did not further investigate physical activity, BMI and ETS. We did not include gene groups and FEV_1_ in our main model since these variables were only available for some participants. After testing for nonlinear effects, we included age as a quadratic term. We identified an interaction between age and sex in our main model and, to facilitate interpretation, we *a posteriori* stratified our main model into children (<14 years) and adults/adolescents (≥14 years). We chose this cut-off due to the hormonal changes during this period, which influence chronic lung disease outcomes [18], and because parents completed the questionnaires for individuals younger than 14 years. Age-stratified models included the following groups – children: <7 years, 7–13 years; adolescents/adults: 14–20 years, 21–40 years, 41–60 years, >60 years.

In subgroups with available data, we analysed effects of FEV_1_ and affected genes. Two sensitivity analyses tested the robustness of our regression model by using the number of exacerbations instead of weeks of exacerbation as the outcome, and by excluding participants who completed >10 questionnaires and reported exacerbations in >50% of these questionnaires. This reduced the influence of a few participants with severe disease and many exacerbations. In a *post hoc* analysis we explored the higher incidence in adult females by testing the hypothesis of disease transmission from child to mother by including “living with a child” as a predictor.

We performed all analysis in R statistical software, version 4.4.1 (www.r-project.org).

## Results

**FIGURE 1:**
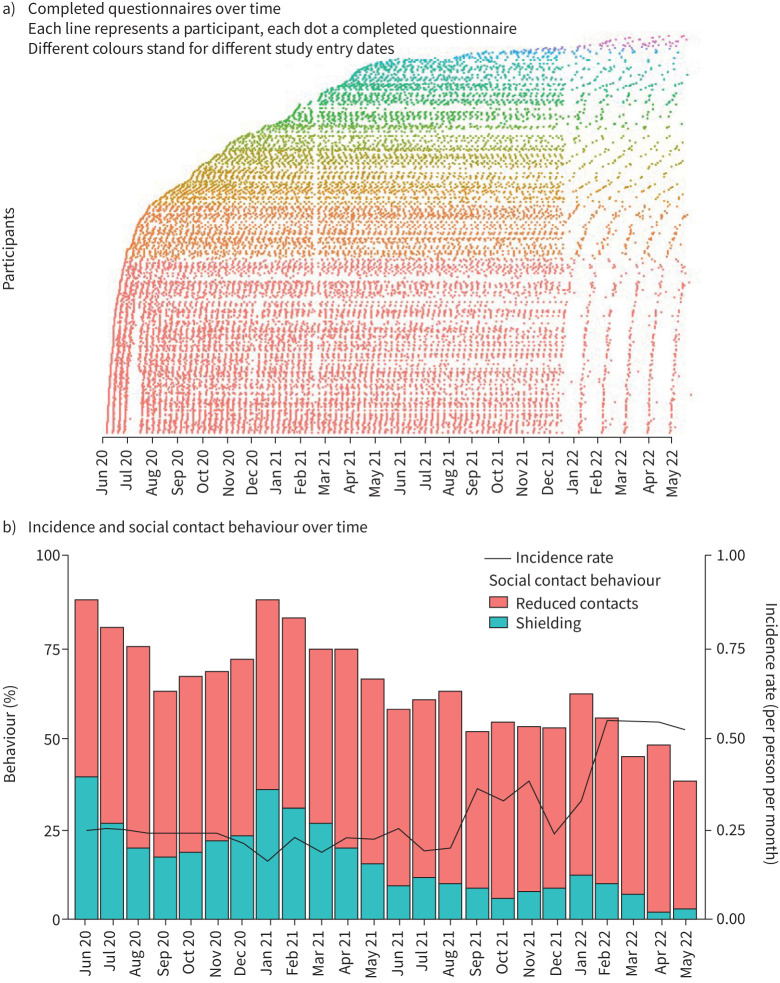
a) Completed questionnaires and b) incidence of pulmonary exacerbations and social contact behaviour over time in the Living with PCD study (n=17 853). PCD: primary ciliary dyskinesia.

We included data from 660 individuals (408 adults, 57 adolescents, 195 children) with at least one follow-up questionnaire completed through May 2022 (table 1). Most were female (399, 60%) with a median age of 28 years (interquartile range (IQR) 12–45) at registration. Participants came from 49 countries, most from North America (139, 21%), the UK (136, 21%) and Germany (99, 15%). 649 (98%) reported having a PCD diagnosis confirmed by a physician, and 11 (2%) had a suspected diagnosis. At registration, 356 of 465 adults and adolescents (77%) and 52 of 195 children (27%) reported bronchiectasis, and 309 (47%) reported prophylactic antibiotics. Participants completed a total of 17 853 follow-up questionnaires between 7 June 2020 and 27 May 2022 (median 17, range 1–84), 40% of questionnaires they received (table 2, figure 1a). We excluded 13 follow-up questionnaires completed after May 2022 or with missing information on symptoms.

**TABLE 1:** Characteristics of people with primary ciliary dyskinesia (PCD) participating in the Living with PCD study, overall and by age group (n=660)

Characteristiccs	Total	Adults/adolescents	Children
**Participants, n**	660	465	195
**Sex**			
Female	399 (60)	314 (68)	85 (44)
Male	259 (39)	149 (32)	110 (56)
Other	2 (0)	2 (0)	0 (0)
**Age years, median (IQR)**	28 (12–45)	38 (26–50)	7 (5–11)
**Age group years**			
<7	87 (13)	-	87 (45)
7–13	108 (16)	-	108 (55)
14–20	57 (9)	57 (12)	-
21–40	207 (31)	207 (45)	-
41–60	162 (25)	162 (35)	-
>60	39 (6)	39 (8)	-
**Country/region^#^**			
North America	139 (21)	94 (20)	45 (23)
UK	136 (21)	97 (21)	39 (20)
Germany	99 (15)	59 (13)	40 (21)
Switzerland	46 (7)	36 (8)	10 (5)
France	40 (6)	27 (6)	13 (7)
Italy	38 (6)	29 (6)	9 (5)
Other European countries	109 (17)	86 (18)	23 (12)
Other non-European countries	53 (8)	37 (8)	16 (8)
**Age at diagnosis years**			
Median (IQR)	7 (2–18)	12 (6–27)	2 (0–6)
Missing	20 (3)	15 (3)	5 (3)
**Congenital heart disease**			
Yes	53 (8)	26 (6)	27 (14)
I don't know	25 (4)	19 (4)	6 (3)
**Bronchiectasis**			
Yes	408 (62)	356 (77)	52 (27)
I don't know	58 (9)	39 (8)	19 (10)
**Prophylactic antibiotics, yes**	309 (47)	215 (46)	94 (48)
**Regular physiotherapy**			
Yes	458 (69)	296 (64)	162 (83)
Missing	4 (1)	3 (1)	1 (1)
**Gene groups** ^¶^	n=186	n=106	n=80
DS	117 (63)	68 (64)	49 (61)
DA	18 (10)	11 (10)	7 (9)
N-DRC	33 (18)	17 (16)	16 (20)
RS-CC	16 (9)	9 (8)	7 (9)
Other	2 (1)	1 (1)	1 (1)
**FEV_1_**	n=497	n=371	n=126
≥60% predicted	308 (62)	223 (60)	85 (67)
<60% predicted	86 (17)	83 (22)	3 (2)
I don't know	103 (21)	65 (18)	38 (30)
***Pseudomonas aeruginosa* isolation, yes**	148 (22)	131 (28)	17 (9)

All characteristics are presented as n (%), unless otherwise stated. DS: dynein structure; DA: dynein assembly; N-DRC: microtubular stabilisation/nexin-dynein regulatory complex; RS-CC: radial spoke and central complex; FEV_1_: forced expiratory volume in 1 s. ^#^: countries or regions with n≥30 displayed in table; countries with n<30 were categorised into other European or other non-European countries. ^¶^: we grouped affected genes according to their role in cilia structure and function, as done previously [25]. DS: *DNAH5* (n=69), *DNAH11* (n=22), *DNAI1* (n=15), *ODAD1* (n=4), *DNAI2* (n=3), *ARMC4* (n=2), *DNAL1* (n=1), *DNAH9* (n=1); DA: *SPAG1* (n=4), *DNAAF4* (n=3), *LRRC6* (n=3), *ZMYND10* (n=2), *DNAAF5* (n=2), *PIH1D3* (*DNAAF6*) (n=2), *CCDC103* (n=1), *CFAP300* (n=1); N-DRC: *CCDC40* (n=17), *CCDC39* (n=12), *CCDC65* (n=4); RS-CC: *RSPH1* (n=7), *HYDIN* (n=5), *RSPH4A* (n=2), *RSPH9* (n=2); other: *RPGR* (n=1), CCNO (n=1).

**TABLE 2:** Pulmonary exacerbation incidence and duration in the Living with PCD study, by age group (n**=**17 853)

	Total	Adults/adolescents	Children
**Number of completed follow-up questionnaires**	17 853	12 915	4938
**Number of questionnaires per person, median (IQR)**	17 (5–46)	17 (5–49)	15 (6–45)
**Person-years at risk^#^**	335	242	93
**Number of exacerbation weeks** ^¶^	1404	1057	347
**Number of exacerbations^+^**	1026	752	274
**Incidence rate per person per year** ^§^	3.06	3.11	2.95
**Duration of an exacerbation (weeks), median (IQR), range** ^ƒ^	1 (1–1), 1–9	1 (1–1), 1–8	1 (1–1), 1–9
**% of exacerbation weeks^##^**	7.9%	8.2%	7.0%

PCD: primary ciliary dyskinesia. ^#^: we calculated person-years at risk by multiplying the number of completed questionnaires outside exacerbations by 7 days, then dividing the result by 365.25 to express it as a yearly measure. ^¶^: number of exacerbation weeks corresponds to the number of weeks during which the BEAT-PCD definition of an exacerbation was met. ^+^: we only counted a new exacerbation if no exacerbation was reported in the last 20 days or if the participant completed a questionnaire indicating a week without an exacerbation since the last exacerbation week. ^§^: we calculated incidence rate by dividing number of exacerbations by person-years at risk. ^ƒ^: we assessed the duration of an exacerbation as the number of consecutive exacerbation weeks. ^##^: we calculated the proportion of exacerbation weeks in all follow-up weeks by dividing the number of exacerbation weeks by the number of completed follow-up questionnaires.

### Incidence of pulmonary exacerbations

Overall incidence was 3.06 pulmonary exacerbations per person per year with only minor variation across age groups (3.11 for adults and adolescents, 2.95 for children), but higher among women (3.70) compared to men (2.00). This rate was based on a total of 1026 exacerbations occurring during 335 person-years at risk (table 2). 235 exacerbations lasted longer than 1 week, resulting in 1404 of 17 853 follow-up weeks (7.9%) meeting the definition of an exacerbation week.

Exacerbation rates changed over the study period, with reduced social interactions corresponding with lower exacerbation rates independent of season (figure 1b). Incidence rates ranged between 0.16 and 0.55 per person per month in the 2 years of follow-up. From spring/summer 2021, we observed an increase in incidence of exacerbations, with an ongoing reduction in shielding behaviour and increased social contacts. Sensitivity analyses with different definitions for exacerbations, time at risk and restricting the sample to people with physician-confirmed diagnoses yielded consistent incidence rates (supplementary table S4).

### Predictors of higher incidence of pulmonary exacerbations

**FIGURE 2:**
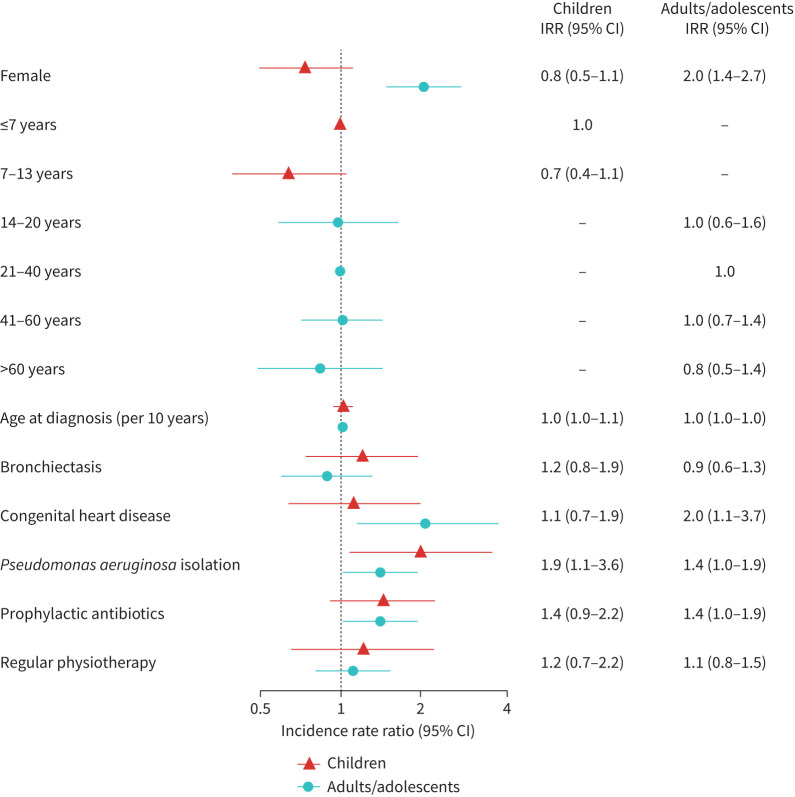
Potential predictors for higher exacerbation incidence in the Living with PCD study, by children (n=189) and adults/adolescents (n=445). We studied predictors of higher incidence of pulmonary exacerbations using negative binomial regression analysis with number of exacerbation weeks as outcome and number of completed questionnaires as offset. We excluded two participants with sex “other”, 20 participants with missing age at diagnosis and four participants with missing physiotherapy from the models. We treated the response “I don't know” for bronchiectasis (n=58) and congenital heart disease (n=25) as “no”. PCD: primary ciliary dyskinesia; IRR: incidence rate ratio.

The fully adjusted multivariable negative binomial regression analysis suggested that people in whom *P. aeruginosa* was identified and those taking prophylactic antibiotics had more exacerbations (table 3). Age, age at diagnosis and bronchiectasis were not associated with incidence. Since we identified an interaction between sex and age, we stratified the main model by age and created separate models for adults and children. In the adult-only model, the incidence among adult females was twice that of adult males (incidence rate ratio (IRR) 2.0, 95% confidence interval (CI) 1.4–2.7) (figure 2). Congenital heart disease and *P. aeruginosa* isolation were also associated with higher incidence among adults (congenital heart disease: IRR 2.0, 95% CI 1.1–3.7; *P. aeruginosa*: IRR 1.4, 95% CI 1.0–1.9). *P. aeruginosa* was a predictor for higher incidence also among children (IRR 1.9, 95% CI 1.1–3.6).

**TABLE 3:** Potential predictors for higher exacerbation incidence among people with primary ciliary dyskinesia (PCD) included in the Living with PCD study (n=660)

	Unadjusted (n=660) IRR (95% CI)	p-value	Fully adjusted (n=634) IRR (95% CI)	p-value
**Sex female (ref.: male)^#^**	1.4 (1.1–1.8)	0.003	0.9 (0.6–1.3)	0.478
**Age, per year**	1.00 (1.00–1.01)	0.394	1.00 (0.98–1.03)	0.711
**Age×age per year^2^**	1.00 (1.00–1.00)	0.800	1.00 (1.00–1.00)	0.064
**Sex×age per year**	1.01 (1.00–1.03)	0.014	1.02 (1.00–1.03)	0.006
**Age at diagnosis,**^¶^ **per 10 years**	1.04 (0.96–1.1)	0.272	1.1 (0.99–1.2)	0.062
**Bronchiectasis Yes (ref.: No)^+^**	1.3 (1.04–1.7)	0.019	1.01 (0.8–1.4)	0.953
**Congenital heart disease Yes (ref.: No)^+^**	1.4 (0.9–2.0)	0.144	1.6 (1.1–2.4)	0.026
***Pseudomonas aeruginosa* isolation Yes (ref.: No)**	1.7 (1.3–2.1)	<0.001	1.5 (1.1–1.9)	0.004
**Prophylactic antibiotics Yes (ref.: No)**	1.6 (1.3–2.0)	<0.001	1.4 (1.1–1.7)	0.011
**Regular physiotherapy Yes (ref.: No)** ^¶^	1.2 (0.95–1.6)	0.095	1.02 (0.8–1.4)	0.560

We studied predictors of higher incidence of pulmonary exacerbations using negative binomial regression analysis with number of exacerbation weeks as outcome and number of completed questionnaires as offset. The fully adjusted model included all listed predictors. IRR: incidence rate ratio. ^#^: we excluded two participants with sex “other” from the model; ^¶^: we excluded 20 participants with missing age at diagnosis and four participants with missing physiotherapy from the model; ^+^: we treated the response “I don't know” for bronchiectasis (n=58) and congenital heart disease (n=25) as “no”.

In subgroups, we also analysed effects of FEV_1_ and affected genes on pulmonary exacerbation incidence but found no associations (supplementary table S5). To explore the higher incidence in adult females, we conducted a *post hoc* analysis that included “living with a child” as a predictor, but found no association. In sensitivity analyses, the findings from our main model stayed consistent if we used number of exacerbations as outcome instead of exacerbation weeks or if we excluded participants with very frequent exacerbations (supplementary table S5).

### Healthcare use, treatment changes and pathogens

Participants contacted a healthcare professional by phone during 758 of 1404 exacerbation weeks (54%) and had an appointment with a healthcare professional during 185 (13%) exacerbation weeks. Participants often managed the exacerbation themselves by changing therapies (1166 exacerbation weeks, 83%), such as starting antibiotic treatment (829 weeks, 59%), increasing physiotherapy (630 weeks, 45%) or increasing inhaler use (455 weeks, 32%) (table 4).

**TABLE 4:** Healthcare use and change of therapies during exacerbations in the Living with PCD study, by adults/adolescents and children

During exacerbations	Overall	Adults/ adolescents	Children
**Participants, n**	1404	1057	347
**Healthcare use**	789 (56)	560 (53)	229 (66)
Contact with health professional by phone due to symptoms	758 (54)	545 (52)	213 (61)
Appointment with health professional due to symptoms	185 (13)	124 (12)	61 (18)
**Change of therapies**	1166 (83)	861 (81)	305 (89)
Start of antibiotic treatment	829 (59)	609 (58)	220 (63)
More physiotherapy	630 (45)	470 (44)	160 (46)
More frequent use of inhaler	455 (32)	382 (36)	73 (21)
More nasal rinsing	368 (26)	302 (29)	66 (19)
More physical activity	109 (8)	83 (8)	26 (7)

All characteristics are presented as n (%). PCD: primary ciliary dyskinesia.

During exacerbations, *P. aeruginosa* was the pathogen most frequently identified in both adults (132 of 303 samples, 44%) and children (18 of 118 samples, 15%). Other pathogens were rare in adults, while in children *Aspergillus fumigatus* (13 of 118 samples, 11%) and *Haemophilus influenzae* (12 of 118 samples, 10%) were found with frequency similar to *P. aeruginosa* (table 5). Outside of exacerbations, *P. aeruginosa* remained the most common pathogen in adults, while in children, *H. influenzae* was slightly more prevalent. During the study period, participants reported 2161 COVID-19 tests of which 93 (4%) were positive; half of the positive samples (45, 48%) were reported during an exacerbation.

**TABLE 5:** Reported pathogens and COVID-19 samples during and outside exacerbations in the Living with PCD study, by adults/adolescents and children

	During exacerbations		Outside exacerbations	
	Adults/ adolescents	Children	Adults/ adolescents	Children
**Pathogens**				
Number of samples	303	118	517	401
Pathogens detected				
*Pseudomonas aeruginosa*	132 (44)	18 (15)	153 (30)	28 (7)
*Haemophilus influenzae*	14 (5)	12 (10)	30 (6)	36 (9)
MSSA	9 (3)	10 (8)	33 (6)	33 (8)
MRSA	12 (4)	3 (3)	8 (2)	4 (1)
*Streptococcus pneumoniae*	8 (3)	8 (7)	14 (3)	14 (3)
*Candida* species	17 (6)	10 (8)	32 (6)	15 (4)
*Aspergillus fumigatus*	11 (4)	13 (11)	28 (5)	11 (3)
**COVID-19**				
Number of COVID-19 samples	290	116	1231	524
COVID-19 positive	24 (8)	21 (18)	26 (2)	22 (4)

All characteristics are presented as n (%). PCD: primary ciliary dyskinesia; MSSA: methicillin-susceptible *Staphylococcus aureus*; MRSA: methicillin-resistant *S. aureus*; COVID-19: coronavirus disease 2019.

## Discussion

Using prospective patient-reported data collected regularly over a 2-year period, we estimated the incidence rate of pulmonary exacerbations among people with PCD to be 3.1 per person per year with minimal variation between age groups but with changes over time. *P. aeruginosa* was frequently detected during exacerbations in children and adults and incidence of exacerbations was higher among adult females and participants from whom *P. aeruginosa* was isolated. Participants often managed exacerbations themselves without visiting healthcare professionals.

Our study is one of few to use prospective data on the incidence of pulmonary exacerbations. We collected weekly information on symptoms and healthcare use, and our comprehensive baseline questionnaire allowed investigating several predictors. Our study preserved participant anonymity, thus, self-reported data could not be verified with clinical records. However, the standard definition of pulmonary exacerbation in PCD used in research is based on patient-reported measures anyway [1]. Similarly, we relied on self-reported PCD diagnoses, which we were unable to validate against diagnostic results from clinics. Although we asked participants to provide diagnostic information, recall was limited, as we have shown in a previous publication from this study [19]. The completion rate of weekly questionnaires (40%) may be a limitation in estimating the incidence rate since it may have resulted in under-reporting (if not all pulmonary exacerbations were recorded) and over-reporting (if participants were more likely to complete questionnaires when symptomatic). We found that participants who completed more questionnaires had lower incidence rates, which favours an overestimation of the true incidence rate in our study (data not shown). In addition, the changes in pathogen incidence during the COVID-19 pandemic due to reduced social contacts and mask-wearing might affect the generalisability of our results to periods outside the pandemic. The frequency of pathogens transmitted *via* droplet infection, such as *H. influenzae*, was reduced during the pandemic [20], which we also observed in our study (data not shown).

We observed similar incidence rates in our study to those reported in the placebo group of the RCT comparing azithromycin to placebo, where all respiratory exacerbations were recorded [8]. However, as per inclusion criteria of the RCT, none of the patients were colonised with *P. aeruginosa* and none received prophylactic antibiotics but had received at least 30 days of antibiotics prescribed for respiratory tract infections or exacerbations within the preceding 2 years [8]. It is important to note that the RCT used a different definition of pulmonary exacerbation than the one applied in our study. Other definitions also exist, such as those used in adults with bronchiectasis, which rely on clinician-reported treatment changes in addition to symptoms [21]. Direct comparisons across studies using different definitions should therefore be made with caution.

Adult females in our study experienced more exacerbations than males. This is consistent with prior findings in PCD showing higher respiratory morbidity in females [15] and it mirrors other respiratory diseases, such as cystic fibrosis, in which females experience more exacerbations after puberty than males, while the reverse trend is observed in children [22]. In these diseases, hormonal influences on the airway surface liquid have been proposed as a potential mechanism. Higher oestrogen levels after puberty may compromise airway surface liquid dynamics needed for effective mucociliary clearance [23]; however, mucociliary clearance is already compromised in females with PCD. Another consideration is that females may be more self-aware of their symptoms, which could lead to increased reporting of symptoms and more proactive health-seeking behaviour. We also explored whether caring for a child, to which mothers usually have closer contact than fathers, contributes to the explanation. We were not able to confirm this hypothesis; however, our data set for analysis was rather small and lacked power. Genetic, morphological, immunological, environmental and lifestyle-related factors can also contribute to sex differences.

*P. aeruginosa* isolation was strongly associated in our analysis with pulmonary exacerbation incidence, which is consistent with previous studies linking *P. aeruginosa* colonisation with poor lung outcomes [9, 15, 24]. However, the presence of *P. aeruginosa* may be less a predictor of exacerbations than it is a marker of advanced lung disease. As microbiology data were self-reported, they may be incomplete or subject to recall bias, with participants more likely to report well-known pathogens such as *P. aeruginosa* that are perceived to negatively impact lung health. Additionally, individuals with more frequent exacerbations are often monitored more closely, including more frequent microbiological testing, which may increase the likelihood of pathogen detection. This increased surveillance may contribute to an overestimation of the observed associations between *P. aeruginosa* isolation and exacerbation incidence. Contrary to intuition, our main model found no significant association between bronchiectasis and pulmonary exacerbations. We cannot know if the effect of bronchiectasis may have been obscured by unmeasured confounders. The observed association between congenital heart disease and higher exacerbation rates in adults may reflect the type or severity of the defect, or long-term haemodynamic consequences such as pulmonary hypertension. Finally, we found an association of prophylactic antibiotics with more exacerbations, which likely reflects confounding by indication. Patients receiving prophylactic antibiotics have more severe disease, which may explain why their exacerbation rates remain high despite treatment. We were unable to detect an association between exacerbations and genotype in the present study, possibly due to the limited sample size, as only a minority of participants reported genotype information (28%). The lack of seasonality in the exacerbation incidence combined with the dependence on social contact behaviour suggests that the anecdotally reported higher incidence in the winter months outside the pandemic may be due to changed social contact behaviour rather than environmental factors such as temperature, humidity or heating use. This complexity of causal relationships between infections, lung damage, therapeutic interventions and environmental factors highlights the need for further prospective studies to better understand the differentiating factors leading to exacerbations.

A notable finding of this study is the low number of patients who reported having seen a healthcare professional during an exacerbation. This may in part reflect the impact of COVID-19 restrictions limiting access to care for non-urgent or less severe cases. In addition, many patients with PCD, particularly those with long-standing diagnoses, may have an exacerbation action plan and a prescription for an antibiotic at home and feel confident to self-manage exacerbations, especially when symptoms are mild. As the BEAT-PCD definition does not capture the severity of exacerbations, some episodes may not have warranted a clinical visit. While self-management promotes patient autonomy, it can also be risky as some patients may underestimate the seriousness of their condition. The consensus definition for pulmonary exacerbations [1] has not yet been validated, and it remains uncertain whether it fully captures true exacerbations. It does not distinguish between more and less severe exacerbations and does not consider the duration of symptoms. In young children, exacerbations are often due to colds, *i.e.* viral infections that are also common in this age group in the general population. In addition, it may be difficult for people with severe illness who do not recover to baseline to accurately report increased symptoms because they cannot clearly distinguish between periods of exacerbation and stability. The definition needs to be validated to ensure the significance of future research on pulmonary exacerbations.

In conclusion, this study confirms that pulmonary exacerbations are frequent in PCD and contribute to high disease morbidity. Adult females and those in whom *P. aeruginosa* can be identified are at particular risk, highlighting the importance of targeted management in these groups.

## Data Availability

Living with PCD data are available upon reasonable request by contacting C.E. Kuehni (claudia.kuehni@unibe.ch).

## Supplementary material

**Please note:** supplementary material is not edited by the Editorial Office, and is uploaded as it has been supplied by the author. Supplementary material 00686-2025.SUPPLEMENT
