# Supplementary material for: Incidence and predictors of self-reported pulmonary exacerbations in primary ciliary dyskinesia: an international prospective cohort study
Source: ERJ Open Res. 2026 Jan 19;12(1):00686-2025. doi: 10.1183/23120541.00686-2025 (PMC12813679; doi:10.1183/23120541.00686-2025)
Supplement: Supplementary file 1 [file 00686-2025.SUPPLEMENT.pdf]

Supplementary Table S1: Formulation of questions and answers related to diagnostic testing from the adult baseline questionnaire in the *Living with PCD* study

Question	Answer
**Have you had diagnostic tests for PCD?**	No<br>Yes
**Have you had a nasal nitric oxide test? (This test measures a gas from the nose through a thin tube that leads to a computer)**	No<br>Yes<br>I don't know/I cannot remember
**What was the nasal nitric oxide test result?**	Normal<br>Suggestive of PCD (very low)<br>Borderline/unclear<br>I don't know/I cannot remember
**Have you had a nasal brush biopsy? (Uncomfortable scraping or brushing to collect cilia/hair cells from the nose, or a brushing to collect cells from the airways during a bronchoscopy)**	No<br>Yes<br>I don't know/I cannot remember
**Do you know if the sample was tested by high speed video microscopy? (The sample was looked at under a microscope to see how the cilia/hairs move)**	No, it was not tested by high speed video microscopy<br>Yes, it was tested by high speed video microscopy<br>I don't know/ I cannot remember
**What was the result of the high speed video microscopy?**	Static, slow, or abnormal movement, typical for PCD<br>Normal movement<br>Unclear result<br>I don't know/ I cannot remember
**Do you know if the sample was tested by electron microscopy? (The sample was looked at with a microscope to see the inside structure of the cilia/hairs)**	No, it was not tested by electron microscopy<br>Yes, it was tested by electron microscopy<br>I don't know/ I cannot remember
**What was the result of the electron microscopy?**	Typical for PCD<br>Normal<br>Unclear result<br>I don't know/I cannot remember
**Have you had a genetic test (looking for genes that cause PCD)?**	No<br>Yes<br>I don't know/ I cannot remember
**Were any genes found that cause PCD?**	No<br>Yes<br>I don't know/ I cannot remember/ waiting for results

Supplementary Table S2: Overview of the items of the BEAT-PCD exacerbation definition with corresponding questions and answer categories in the *Living with PCD* follow-up questionnaires, including the number of questionnaires in which the answer category was ticked by the participants **(n = 17,853)**

Items of the BEAT-PCD exacerbation definition	Corresponding question in the *Living with PCD* follow-up questionnaire	Answer categories in the *Living with PCD* follow-up questionnaire	Number of questionnaires (n = 17,853)
**Increased cough**	Did you have any cough in the last 7 days?	No, I did not cough	1780
		**Yes, I coughed more than usual**	**2016**
		Yes, but I coughed no more than usual	13470
		Yes, but I coughed less than usual	526
		Missing	61
**Increased cough reported in 2016 questionnaires**			
**Change in sputum volume and/or colour**	Did you cough up any mucus (including swallowing it) in the last 7 days?	No, I coughed up no mucus	1628
		**Yes, I coughed up more mucus than usual**	**1903**
		Yes, but I coughed up the same amount as usual	13468
		Yes, but I coughed up less mucus than usual	800
		Missing	54
	Was the colour of the mucus different than it usually is?	No	12780
		**Yes**	**1348**
		I don't know	1182
		Missing	115
**Change in sputum volume and/or colour reported in 2499 questionnaires**			
**Increased shortness of breath perceived by the patient or parent**	Did you have any shortness of breath in the last 7 days?	No, I did not have shortness of breath	9289
		**Yes, I had more shortness of breath than usual**	**1485**
		Yes, but I had as much shortness of breath as usual	6809
		Yes, but I had less shortness of breath than usual	222
		Missing	48
**Increased shortness of breath reported in 1485 questionnaires**			
**Decision to start or change antibiotic treatment because of perceived pulmonary symptoms**	Which advice did you get from your health professional?	Wait and see	256
		**Start antibiotics treatment**	**649**
		Increase physiotherapy/respiratory therapy	316
		See a health professional	110
		Go to the hospital	154
		**Other advice**	224
		**[start/change of antibiotics treatment mentioned in comment box]**	**15**
	Have you taken any prescribed or over-the-counter medication against the symptoms?	No medication taken	2091
		Yes, fever medication/painkillers (e.g. paracetamol, acetaminophen, aspirin, ibuprofen)	1438
		Yes, expectorant to loosen mucus	563
		Yes, antiviral medication (e.g. Tamiflu, Relenza)	36

		**Yes, antibiotics**	**1059**
		**Yes, other**	499
		**[start/change of antibiotics treatment mentioned in comment box]**	**16**
		I don't know/can't remember	11
**Decision to start or change antibiotic treatment reported in 1189 questionnaires**			

**Malaise, tiredness, fatigue or lethargy**	Did you notice a worsening or new occurrence of the symptoms listed below in the last 7 days?	Tiredness or exhaustion	**1293**
**Malaise, tiredness, fatigue or lethargy reported in 1293 questionnaires**			

**New or increased haemoptysis**	What was the colour of the mucus?	Mixed with blood	**202**
**New or increased haemoptysis reported in 202 questionnaires**			

**Temperature > 38°C**	Did you have any fever/temperature in the last 7 days?	No	16840
		**Yes**	**798**
		I don’t know	164
		Missing	51
**Fever reported in 798 questionnaires**			

Supplementary Table S3: Potential predictors for higher exacerbation incidence and other variables of interest in the *Living with PCD* study

Variable	Definition	Questionnaire
**Predictors**		
**Sex**	We categorised sex as “male”, “female”, or “other”. For the regression analysis, we excluded participants with sex “other”.	Baseline
**Age**	We used age at registration and included it either as a continuous variable or categorised into age groups; < 7 years, 7-13 years, 14-20 years, 21-40 years, 41-60 years, > 60 years.	Baseline
**Country/region**	We categorised countries or regions with N<30 into other European or other non-European countries.	Baseline
**Age at diagnosis**	We assessed age at PCD diagnosis, included as continuous variable. We excluded participants with missing age at diagnosis from the regression models.	Baseline
**Congenital heart disease**	We categorised the presence of congenital heart disease as “yes”, “no”, or “I don’t know”, with the latter grouped with “no” for regression analysis.	Baseline
**Bronchiectasis**	We categorised the presence of bronchiectasis as “yes”, “no”, or “I don’t know”, with the latter grouped with “no” for regression analysis.	Baseline
**Prophylactic antibiotics**	We assessed the regular use of antibiotics to prevent bacterial infections and categorised it into “yes” and “no”.	Baseline
**Regular physiotherapy**	We assessed the frequency of respiratory physiotherapy/airway clearance in the last month and categorised it into *regular* (several times a week, daily, twice daily or more) and *irregular* (less than once a week/only when I was not well, never). We excluded participants with missing physiotherapy from the regression analysis.	Baseline
**Gene groups**	We grouped affected genes according to their role in cilia structure and function (dynein assembly, dynein structure, microtubular stabilisation/nexin-dynein regulatory complex, radial spoke and central complex, other), as done previously (Shoemark et al., 2021). We excluded the group “other” from the regression analysis.	Baseline
**FEV1**	We categorised FEV1 as ≥ 60% predicted, < 60% predicted, and “I don’t know”, with the latter and missing values excluded from the regression analysis.	Baseline
***Pseudomonas aeruginosa* isolation**	We classified participants as “*Pseudomonas aeruginosa* isolated” if they tested positive for *Pseudomonas aeruginosa* in the 12 months before study enrolment.	Baseline
**Social contact behaviour**		
**Reduced contacts**	We defined reduced contacts if they reported having contact with less than 10 people in the past week but were not shielding.	Follow-up
**Shielding**	We defined shielding if participants reported no contact with anyone in the last week or only with those they live with.	Follow-up
**Healthcare use**		
**Contact with health professional by phone**	We defined contact with a health professional by phone if participants contacted any health professional due to increased symptoms in the last 7 days.	Follow-up
**Appointment with health professional**	We defined an appointment with a health professional if they saw any health professional due to increased symptoms in the last 7 days.	Follow-up
**Change of therapies**	We defined change of therapies if participants increased or changed any of their usual therapies in the last 7 days.	Follow-up

Supplementary Table S4: Incidence rate estimated by the main analysis, compared to results obtained by alternative approaches to modelling (sensitivity analyses) with a) an alternative calculation of time at risk (including all completed questionnaires) and with a different time span of b) 30 days and c) 14 days between exacerbations, and d) only including participants with a physician-confirmed diagnosis, in the *Living with PCD* study.

	**Main model**	Sensitivity analyses			
		a	b	c	d
	**Total**	Total	Total	Total	Total
Number of completed follow-up questionnaires	**17,853**	17,853	17,853	17,853	**17,542**
Person-years (at risk)	**335**	**342**	335	335	**329**
Number of exacerbations	**1026**	1026	**990**	**1066**	**1007**
Incidence rate (per person per year)	**3.06**	3.00	2.96	3.18	**3.06**

Abbreviation: PCD, primary ciliary dyskinesia.

Supplementary Table S5: Subgroup, post hoc and sensitivity analyses: Incidence rate ratios of pulmonary exacerbations from a negative binomial regression analysis among people with primary ciliary dyskinesia included in the *Living with PCD* study with a) FEV1 included as a predictor (N = 379), b) gene groups included as a predictor (N = 182), c) "living with a child" included as a predictor in a model which only included adult females (N = 300), d) number of exacerbations as outcome (N = 638), and e) people who reported exacerbations in more than 50% of questionnaires excluded (N = 633).

		**a**<br>FEV1 included as a predictor<br>N = 375<br>IRR (95% CI)	**b**<br>Gene groups included<br>N = 182<br>IRR (95% CI)	**c**<br>"Living with a child" included, only adult females<br>N = 298<br>IRR (95% CI)	**d**<br>Exacerbation as outcome<br>N = 634<br>IRR (95% CI)	**e**<br>People with exacerbation in > 50% of questionnaires excluded<br>N = 629<br>IRR (95% CI)
**Sex** (ref.: Male)[a]	Female	0.7 (0.4-1.3)	0.8 (0.5-1.5)		0.9 (0.6-1.4)	0.9 (0.6-1.4)
**Age**	Per y	1.0 (1.0-1.1)	1.0 (1.0-1.0)	1.0 (1.0-1.1)	1.0 (1.0-1.0)	1.0 (1.0-1.0)
**Age * age**	Per y^2	1.0 (1.0-1.0)	1.0 (1.0-1.0)	1.0 (1.0-1.0)	1.0 (1.0-1.0)	1.0 (1.0-1.0)
**Sex * age** (ref.: Male)	Per y	1.0 (1.0-1.0)	1.0 (1.0-1.0)		1.0 (1.0-1.0)	1.0 (1.0-1.0)
**Age at diagnosis**[a]	Per 10 y	1.2 (1.1-1.4)	1.0 (0.8-1.2)	1.2 (1.0-1.3)	1.1 (1.0-1.2)	1.1 (1.0-1.2)
**Bronchiectasis** (ref.: No)[b]	Yes	0.9 (0.6-1.2)	1.0 (0.6-1.7)	1.2 (0.7-1.8)	1.0 (0.8-1.3)	1.1 (0.8-1.4)
**Congenital heart disease** (ref.: No)[b]	Yes	1.6 (1.0-2.8)	1.8 (1.0-3.1)	2.2 (1.1-4.8)	1.4 (1.0-2.0)	1.5 (1.0-2.2)
**P. aeruginosa isolation** (ref.: No)	Yes	1.3 (1.0-1.8)	2.0 (1.3-3.2)	1.5 (1.1-2.2)	1.4 (1.1-1.7)	1.4 (1.1-1.9)
**Prophylactic antibiotics** (ref.: No)	Yes	1.3 (1.0-1.8)	1.1 (0.7-1.6)	1.3 (0.9-1.8)	1.2 (0.9-1.5)	1.2 (1.0-1.6)
**Regular physiotherapy** (ref.: No)[a]	Yes	1.1 (0.8-1.6)	1.4 (0.8-2.4)	0.8 (0.6-1.2)	1.1 (0.9-1.4)	1.3 (1.0-1.6)
**Gene groups** (ref.: DS)[a]	DA		1.0 (0.5-1.9)			
	N-DRC		0.7 (0.4-1.1)			
	RS-CC		1.3 (0.7-2.5)			
**FEV1** (ref.: ≥ 60%)[a]	< 60%	1.3 (0.9-1.9)				
**Living with a child** (ref.: No)	Yes			1.1 (0.7-1.8)		

Abbreviations: DA, dynein assembly. DS, dynein structure. CI, confidence interval. FEV1, Forced expiratory volume in the first second. IRR, incidence rate ratio. N-DRC, microtubular stabilisation/nexin-dynein regulatory complex. P. aeruginosa, Pseudomonas aeruginosa. PCD, primary ciliary dyskinesia. ref, reference. RS-CC, radial spoke and central complex. y, year. [a] We excluded participants with sex "other", missing age at diagnosis, missing physiotherapy, gene group "other", and unknown FEV1 from the respective models. [b] We treated the response "I don't know" for bronchiectasis (n = 58) and congenital heart disease (n = 25) as "no". We present IRR and 95% CI. DS: *DNAH5* (n=69), *DNAH11* (n=22), *DNAI1* (n=15), *ODAD1* (n=4), *DNAI2* (n=3), *ARMC4* (n=2), *DNAL1* (n=1), *DNAH9* (n=1); DA: *SPAG1* (n=4), *DNAAF4* (n=3), *LRRC6* (n=3), *ZMYND10* (n=2), *DNAAF5* (n=2), *PIH1D3* (*DNAAF6*) (n=2), *CCDC103* (n=1), *CFAP300* (n=1);

N-DRC: *CCDC40* (n=17), *CCDC39* (n=12), *CCDC65* (n=4); RS-CC: *RSPH1* (n=7), *HYDIN* (n=5), *RSPH4A* (n=2), *RSPH9* (n=2).
